# Assessment of wound area reduction on chronic wounds in dogs with photobiomodulation therapy: A randomized controlled clinical trial

**DOI:** 10.14202/vetworld.2021.2251-2259

**Published:** 2021-08-28

**Authors:** Somphong Hoisang, Naruepon Kampa, Suvaluk Seesupa, Supranee Jitpean

**Affiliations:** 1 Veterinary Teaching Hospital, Faculty of Veterinary Medicine, Khon Kaen University, Khon Kaen, Thailand; 2 Division of Surgery, Faculty of Veterinary Medicine, Khon Kaen University, Khon Kaen, Thailand

**Keywords:** canine, chronic wound, combined wavelength photobiomodulation therapy, low-intensity laser therapy, photobiomodulation therapy

## Abstract

**Background and Aim:**

Chronic wounds are a clinical problem and require intensive standard wound care. However, this is sometimes insufficient to promote healing. Photobiomodulation therapy (PBMT) can be used as an adjunctive therapy to improve wound healing. Various PBMT devices with different properties and parameter settings as well as different animal species can influence a variety of clinical outcomes. This study aims to assess the use of 830 nm PBMT or simultaneous superpulsed and multiple wavelengths (SPMW; 660, 875, and 905 nm) PBMT on chronic wounds in client-owned dogs.

**Materials and Methods:**

This study included 21 client-owned dogs with chronic wounds allocated into three groups: (1) Control group (C) treated with irrigated saline and without PBMT (n=7); (2) L1 group treated with irrigated saline together with the radiation of 830 nm PBMT (n=7); and (3) L2 group treated with irrigated saline together with the radiation of simultaneous SPMW-PBMT (n=7). Wound healing was assessed on the basis of wound size reduction as a percentage of wound area every 2^nd^ day for 15 days using image analysis software (ImageJ software^®^, National Institutes of Health, Rockville, Maryland, USA).

**Results:**

A significant difference in the percentage of wound area reduction was noted between the C and PBMT groups (L1 and L2; p<0.05). The average percentages of wound area reduction at the end of the study (15 days) were 42.39±20.58, 56.98±24.82, and 61.81±27.18 in the C, L1, and L2 groups, respectively. A steady decrease in wound size was noted in both PBMT and non-PBMT groups, and coefficients were 7.77, 8.95, and 10.01 in the C, L1, and L2 groups, respectively. The percentage of wound area reduction was found to be significantly different between the PBMT and non-BPMT groups on day 7 (p<0.05).

**Conclusion:**

Based on the results of the current study, using either 830 nm PBMT or simultaneous SPMW-PBMT can accelerate the chronic wound healing process in dogs with a significant reduction in wound area. Therefore, it can be used as an adjunctive therapy to improve wound healing in dogs with reduced treatment duration.

## Introduction

A chronic wound is defined as a wound that fails to heal over 3 weeks with standard wound care [1,2]. Moreover, chronic wounds require intensive standard wound care; however, this is sometimes insufficient to promote healing. At present, photobiomodulation therapy (PBMT) can be used as adjunctive therapy for improving wound healing in veterinary medicine [3,4], dentistry [5], and medicine [6,7]. PBMT involves the use of therapeutic light, including laser, light-emitting diode, and broadband light, in the visible and infrared spectrum [8]. The photobiomodulation process is well understood where it can stimulate endogenous chromophores in mitochondrial membranes and produce cellular adenosine triphosphate, which is a universal fuel inside living cells [9]. It stimulates the mitochondrial membrane potential, leading to the release of nitric oxide, and modulates reactive oxygen species as a photochemical reaction, which drives all biological reactions and improves cellular metabolism [10]. PBMT has been reported to influence various phases of wound healing [11,12], including increased endothelial cells and keratinocyte proliferation, fibroblast proliferation, collagen deposition, angiogenesis, granulation tissue formation, and improvement of wound tensile strength [13,14].

A few clinical studies in veterinary medicine have been conducted to evaluate the use of PBMT on open wounds and had various outcomes. The PBMT using a 632.8 nm wavelength with a fluence of 2.64-3.64 J/cm^2^ accelerated wound healing by increasing epithelialization in dairy cattle [15]. The use of a 635 nm wavelength with a fluence of 5.1 J/cm^2^ gave significantly faster equine wound healing from the epithelialization result [4]. Recently, applying a simultaneous combination of 850 and 670 nm wavelengths with a fluence of 8 J/cm^2^ significantly improved the clinical scar scale in patients with thoracolumbar hemilaminectomy [16]. However, several PBMT parameters had no beneficial effects on wound healing. PBMT using 635 and 980 nm wavelength with a fluence of 1.125 [17] and 5 J/cm^2^ [18] did not enhance wound healing of canine incised wound healing by complete granulation tissue formation and epithelialization.

Simultaneous superpulsed and multiple wavelengths (SPMW)-PBMT equipment provides red light (660 nm), broadband light (875 nm), and superpulsed light (905 nm) [19,20], providing a higher peak power and more beneficial effects into the target tissue without thermal effects [21,22]. However, information on simultaneous SPMW-PBMT application as an alternative treatment for wounds in small animals is limited.

This study aims to evaluate the clinical outcome of a single wavelength of 830 nm PBMT, simultaneous SPMW-PBMT (660, 875, and 905 nm), and without PBMT on chronic wounds in client-owned dogs. The hypothesis was that a chronic wound treated with PBMT can heal faster without PBMT as assessed by wound area reduction.

## Materials and Methods

### Ethical approval and Informed consent

This study was a randomized controlled clinical trial and was approved by the Institutional Animals Care and Use Committee of Khon Kaen University (IACUC-KKU 17/61). The owners allowed their dogs to participate and signed the consent form before study commencement.

### Study period and location

This study was conducted from May 2018 to July 2020. All procedures were performed at Veterinary Teaching Hospital (VTH), Khon Kaen University, Thailand.

### Samples

The sample size of the study was calculated based on a study of wound healing comparison using a superiority trial (two-sample parallel design for continuous data) [23]. The effect on the size of wound healing between groups was 7.11, and the standard deviation was 4.94, in combination with a specified significance level of 0.05 and beta probability of 0.2 [24]. Client-owned dogs presented at VTH, Khon Kaen University with a chronic wound that failed to heal after 3 weeks of wound age and had a wound area of at least 4 cm^2^ were recruited into the study. The treatment cost of enrolled dogs was provided from the research fund during the study or the wound was completely healed (2-4 weeks). The dogs were considered healthy based on general physical examinations, complete blood counts, and serum biochemistry analyses. The inclusion/exclusion criteria of the current study are shown in Table-1.

**Table-1:** Inclusion/exclusion criteria of the study.

Inclusion criteria	Exclusion criteria
Chronic wound over 3 weeks	Presence of wound exudate/discharge
Full-thickness open wound area >4 cm^2^ from any cause; vehicle trauma, bite wound, surgical wound dehiscence	Underlying conditions: Anemia, malnutrition, diabetes mellitus, and others that affect wound healing
Bacterial culture found with <10^5^ CFU or no growth within 48 h	Contraindication to PBMT; evidence of bone growth plate, suspected tumors
Availability of written consent	No possibility of wound cover change every 2^nd^ day

PBMT=Photobiomodulation therapy

In this study, all dogs with wounds were initially treated with standard wound care with the same course of antibiotics and analgesics (cephalexin, 22-30 mg/kg twice daily; tramadol hydrochloride, 3-4 mg/kg twice daily; topical nitrofural [Bactacin^®^, Osoth Inter Laboratories, Thailand]). Susceptibility antibiotics were used as needed until signs of wound infection were no longer noted. Dogs with wounds for more than 3 weeks after treatment and without purulent wound discharge or signs of wound infections based on the bacterial culture were considered to be delayed wound healing subjects and were enrolled in this study. Twenty-four dogs met the inclusion criteria. However, three dogs were excluded from the study: The first, second, and third cases presented neurological signs on day 9 of the study, lost contact on day 3 of treatment, and presented with anemia and blood parasite infection, respectively. Therefore, 21 dogs were used in the study (12 male and 9 female dogs; average, 5.3 years [range, 1-12 years old]). The average wound age was 3.9 weeks (range, 3-11 weeks). The wounds were caused by a bite (47.62%), vehicular accident trauma (23.81%), and surgical wound dehiscence (28.57%).

### Treatments

The selected cases were randomly allocated into three groups using the Microsoft Excel program: Control group (C; non-PBMT) treated with irrigated saline and without PBMT, L1 group treated with irrigated saline together with the radiation of 830 nm PBMT, and the L2 group treated with irrigated saline together with the radiation of simultaneous SPMW-PBMT. The wounds in the C group were treated with irrigated saline without topical medication every 2^nd^ day for 2 weeks. The two PBMT groups (L1 and L2) were treated with irrigated saline without topical medication together with the different PBMTs every 2^nd^ day for 2 weeks (Table-2). The wound in the L1 group was radiated with a single wavelength of 830 nm PBMT (BTL-5800 SL Combi, BTL Industries Ltd., London, UK) with the dose setting recommended by Millis and Saunders [25] and based on our previous study [26]. The wound in the L2 group was radiated with simultaneous SPMW-PBMT (660, 875, and 905 nm; MR4 ActiVet Pro Veterinary Laser, Multi Radiance Medical, Solon, OH, USA) with a preset program for tissue repairing protocol from the manufacturer’s recommendations. The PBMT device probe was used with a noncontact technique (1-cm distance wound probe) and was irradiated covering the entire wound with a 0.5-cm margin surrounding the wound. Sedation in any of the dogs was not required for wound care. The wound was covered by a sterile gauze pad and changed every 2^nd^ day. All procedures for each dog were performed by one observer (SH).

**Table-2:** Treatment protocol and PBMT parameter.

Group	Treatment protocol	PBMT parameter	PBMT equipment
C	Irrigated saline lavage without topical medication	-	-
L1	Irrigated saline lavage without topical medication + PBMT	830 nm wavelength with fluence of 4 J/cm^2^, power of 200 mW, and frequency of 50 Hz; time of treatment was calculated from the wound area which is between 3.45 and 41.40 min	BTL-5800 SL Combi, BTL Industries Ltd., UK
L2	Irrigated saline lavage without topical medication + PBMT	Synchronous use of light power of SPMW (100 mW of 660 nm; 250 mW of 875 nm; peak pulse power 50 W [pulse duration of 110±20 s] of 905 nm), time of treatment used was 1 min/4 cm^2^ of wound area which is between 1 and 5 min	MR4 ActiVet Pro, Multi Radiance Medical^®^, USA

PBMT=Photobiomodulation therapy. C: Control group; L1: Adjunctive therapy with the 830 nm wavelength PBMT (BTL-5800 SL Combi); L2: Adjunctive therapy with superpulsed multiple wavelength PBMT (MR4 ActiVet Pro Veterinary Laser)

### Wound healing measurement

Wound healing was assessed by the change of wound size obtained from a wound photograph that was adapted from the Bates–Jensen Wound Assessment [27]. The wounds were photographed using a 12-megapixel digital camera (Olympus E-PM1 with 14-42 II lens, Japan) with a calibrated scale beside the wound edge and inside the photo frame. Photographs were taken perpendicular to the wound and approximately 15 cm from the wound on days 1, 3, 5, 7, 9, 11, 13, and 15 by the same observer (SH). The wound area was calculated by ImageJ software^®^(National Institutes of Health, USA) [12,17,28]. The wound area on the initial day of treatment was considered as 100%. The change of wound size was compared with that of day 1 and was reported as the percentage of wound area reduction. The percentage of wound area reduction was calculated using the previously published formula [29-31]:

% wound area = [(W1-Wx)/W1] X 100

W1= the initial wound area

Wx= the area on measurement day.

### Statistical analysis

The C and experimental (L1 and L2) groups were statistically analyzed by a repeated linear mixed model (two levels) to find the average wound area reduction evaluation. The full model included fixed effects as a treatment protocol, healing day evaluation, and their interaction effect. A random slope was a subject’s (dog’s) response as wound healing evaluation measured on several time points with a covariance structure (unstructured). Statistical analysis was performed using commercially available software, STATA version 10.1 (StataCorp LLC, USA). The level of significance was considered as p<0.05.

## Results

**Figure-1:**
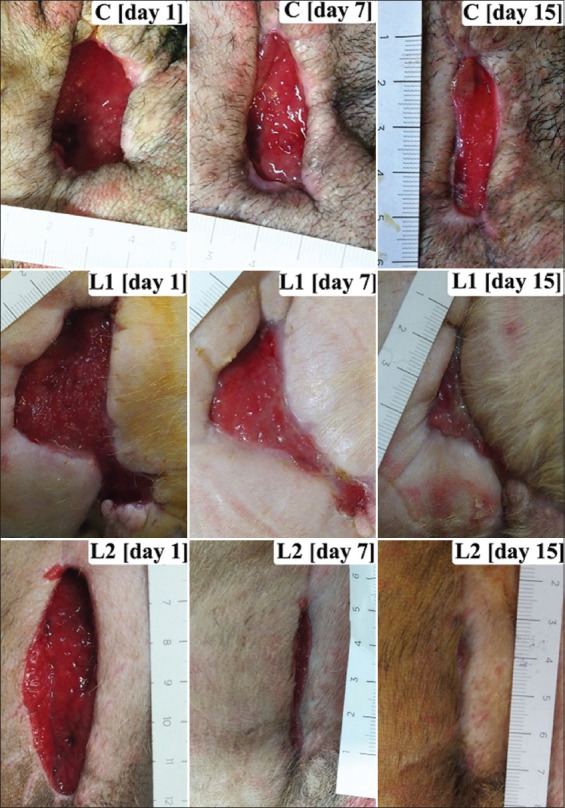
Wound photographs showing the variety of the initial wound area of clinical cases. The percentages of wound area reduction of photobiomodulation therapy (PBMT) groups (L1 and L2) were significantly higher than the non-PBMT group (C) from days 7 to 13 after beginning the treatment (p<0.05).

**Figure-2:**
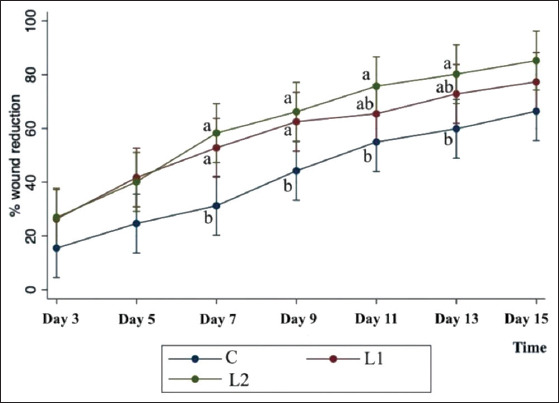
A linear graph of percentage of wound reduction with time, showing a significant increase over time (a lower-case letter indicates a significant difference, p<0.05), the coefficients were 7.77, 8.95, and 10.01 in C, L1, and L2, respectively. In addition, the wound treated with simultaneous superpulsed and multiple wavelengths-photobiomodulation therapy (SPMW-PBMT) was observed to have a tendency to be better than the 830 nm PBMT group and control in the reduction of wound size (C: Control group; L1: Adjunctive therapy with the 830 nm wavelength PBMT; and L2: Adjunctive therapy with simultaneous SPMW-PBMT).

A significant difference in wound size between groups with an average of 21.7 cm^2^ (p<0.05; range, 4.0-104.6 cm^2^) was noted before treatment (Table-3). Therefore, the wound area was adjusted as the percentage of wound area reduction at baseline before data analyses. A steady decrease in wound size was noted in both PBMT and non-PBMT groups (Figure-1). The change in wound size (percentage of wound area reduction) significantly increased over time with the coefficients 7.77, 8.95, and 10.01 for C, L1, and L2, respectively (Figure-2). However, the percentage of wound area reduction was found to be significantly different between PBMT and non-BPMT groups on day 7 (p<0.05). At the end of the study (day 15), the wounds of the two cases in the L2 group were completely healed. Overall, a significant difference in the percentage of wound area reduction was noted between the C and PBMT groups (L1 and L2; p<0.05). The average percentages of wound area reduction were 42.39±20.58, 56.98±24.82, and 61.81±27.18 in C, L1, and L2, respectively (Table-4).

**Table-3:** Summary of dogs in the study; signalment, cause, location, treatments, and results.

Dog	Gender	Age (years)	Cause of wound	Location of wound	Wound age (weeks)	Treatment	Wound area before treatment (cm^2^)	Wound area after 2 weeks of treatment (cm^2^)	Wound area reduction (%)
No. 1	Male	1	Bite wound	Dorsal neck	3	L2	5.61	0.00	100.00
No. 2	Female	5	Bite wound	Ventral neck	3	L2	4.00	0.29	92.62
No. 3	Male	5	Vehicle trauma	Foreleg	4	L2	10.07	4.34	56.90
No. 4	Female	4	Bite wound	Dorsal neck	4	C	4.09	1.10	73.13
No. 5	Female	9	Vehicle trauma	Lateral abdomen	4	C	5.23	1.62	68.92
No. 6	Female	4	Bite wound	Lateral thorax	8	C	13.2	-	Excluded
No. 7	Female	4	Bite wound	Lateral thorax	3	L1	10.88	3.74	65.65
No. 8	Female	5	Bite wound	Lateral thorax	3	L1	104.59	58.96	43.63
No. 9	Female	10	Vehicle trauma	High leg	3	C	7.07	2.03	71.31
No. 10	Male	5	Bite wound	Shoulder	3	C	12.07	2.40	80.12
No. 11	Female	3	Surgical wound dehiscence	Ventral abdomen	4	L2	7.55	0.34	95.50
No. 12	Male	5	Vehicle trauma	High leg	3	C	12.25	5.06	58.69
No. 13	Female	12	Surgical wound dehiscence	High leg	3	L1	9.02	0.06	99.33
No. 14	Male	4	Vehicle trauma	High leg	3	L2	20.15	4.26	78.84
No. 15	Male	5	Bite wound	High leg	3	L1	79.96	18.57	76.77
No. 16	Female	7	Bite wound	Lateral abdomen	3	L2	5.76	-	Excluded
No. 17	Male	3	Bite wound	Shoulder	3	C	46.37	17.68	61.87
No. 18	Female	4	Abscess	Shoulder	3	L2	12.1	-	Excluded
No. 19	Female	3	Bite wound	Shoulder	3	L1	13.9	2.05	85.25
No. 20	Male	12	Bite wound	Foreleg	11	L2	4.01	0.00	100.00
No. 21	Male	1	Surgical wound dehiscence	Ventral abdomen	3	C	6.46	3.18	50.84
No. 22	Male	5	Surgical wound dehiscence	Ventral abdomen	8	L1	26.93	4.46	83.45
No. 23	Male	5	Bite wound	Dorsal neck	3	L1	53.14	6.83	87.15
No. 24	Male	5	Vehicle trauma	Shoulder	4	L2	13.16	3.59	72.75

C: Control group; L1: Adjunctive therapy with the 830 nm wavelength PBMT (BTL-5800 SL Combi); L2: Adjunctive therapy with superpulsed multiple wavelength PBMT (MR4 ActiVet Pro Veterinary Laser). PBMT=Photobiomodulation therapy

**Table-4:** Comparison of the percentage of wound area reduction (mean±SD) between control group (C) and the PBMT-treated groups (L1 and L2) on days 1, 3, 5, 7, 9, 11, 13, and 15.

Group	Day 1	Day 3	Day 5	Day 7	Day 9	Day 11	Day 13	Day 15	Average
C	-	15.43±10.93	24.63±9.33	31.22±7.82	44.22±10.20	54.95±12.70	59.87±13.31	66.41±9.88	42.39±20.58
L1	-	26.28±13.03	41.72±20.11	52.76±19.35	62.50±21.27	65.46±22.39	72.84±20.30	77.32±18.04	56.98±24.82
L2	-	26.88±19.02	40.13±22.08	58.27±20.06	66.20±18.99	75.72±19.84	80.21±19.20	85.23±16.31	61.81±27.18
p-value	-	0.26	0.06	0.00	0.01	0.03	0.03	0.06	0.01

C: Control group; L1: Adjunctive therapy with the 830 nm wavelength PBMT; L2: Adjunctive therapy with superpulsed multiple wavelength PBMT. PBMT=Photobiomodulation therapy

## Discussion

The current study indicated that PBMT can accelerate wound healing assessed on the basis of wound area reduction. The wounds in this study were treated with PBMT as an adjunctive therapy. Moreover, treatment with a single wavelength of 830 nm PBMT with a fluence of 4 J/cm^2^, power of 200 mW, and frequency of 50 Hz, and simultaneous SPMW-PBMT with a preset of 1-250 Hz showed significant improvement of the wound healing process on the basis of wound size reduction compared with a non-PBMT treatment from days 7 to 13 after treatment. This finding supports a previous PBMT parameter study using an 830 nm light with a dose of 4 J/cm^2^ that would be appropriate for superficial wound treatment in dogs [26]. In addition, our result is related to the findings of the previous studies, which noted an improvement in mice wound healing with the use of a single 830 nm PBMT with a fluence of 3-4.2 J/cm^2^ [32,33]. In clinical case reports, human venous ulceration wounds treated with 830 nm PBMT with a dose of 9 J/cm^2^ [34] and chronic dog wounds treated with the use of a single 630 nm wavelength PMBT with a fluence of 5 J/cm^2^ [28] exhibited more rapid wound area reduction. This potentially confirms *in vitro* studies showing that PBMT improved biological immune response by increasing the migration of primary cytokines (interleukin-1β [IL-1β], tumor necrosis factor-a, IL-6, and MCP-1) [35], neutrophil and macrophage infiltration [36], angiogenesis, fibroblast and collagen formation, reepithelialization, and wound tensile strength [37-39].

At present, evidence of the use of simultaneous SPMW-PBMT on wound healing in dogs is limited. Comparing the studies with different PBMT devices and parameter settings are difficult. One study investigated the effect of simultaneous SPMW-PBMT at a preset of 5 Hz and found no apparent improvement on surgically induced full-thickness wounds in amphibians [19]. However, several studies were impressed with the outcome of the combination of wavelength PBMT. Applying a combination of 637 (0.2 J/cm^2^) and 956 (1.2 J/cm^2^) nm, PBMT ­presented a significant decrease in wound size and treatment time in equine wounds [40]. Moreover, a combination of 660 and 890 nm PBMT at a dose of 3 J/cm^2^ can accelerate diabetes leg ulcer wound healing in humans [41]. Similarly, clinical use of 850 and 670 nm and a dose of 8 J/cm^2^ showed significantly improved cosmetic healing of incised wounds in dogs [16].

Interestingly in this study, no statistical difference (p=0.85) was noted when comparing the L1 and L2 groups, although the wounds treated with simultaneous SPMW-PBMT were observed to tend to be better than the 830 nm PBMT-treated group in wound size reduction (Figure-2). A further study needs to be done to confirm this finding. Several studies of potential applications *in vitro* have been done, demonstrating that the use of combined PBMT showed evidence of new blood vessel formation, intense inflammatory reaction, collagen matrix formation, and reepithelization [38,42-44]. Radiation at different wavelengths would affect different target tissues, and the tissue could absorb different amounts of radiation [45]. A synergistic effect based on a combination of red light, infrared, and superpulsed technology may occur in target cells and tissue surrounding those cells directly irradiated [7]. Recent *in vivo* studies of superpulsed light showed a deeper penetration in human [46] and horse tissues [20]. Therefore, the application of simultaneous SPMW-PBMT for the treatment of deep tissue conditions may give better outcomes. Moreover, the advantage of a simultaneous SPMW-PBMT device is the probe, which is designed as a shower probe for delivering synchronous multiple therapeutic light sources. This type of probe would provide more power and reduce treatment time.

Despite the results of the current study confirming that the use of both PBMTs showed positive results on chronic wound healing, several studies have shown no significant difference of PBMT on the healing of surgically created wound in dogs [17,18]. The difference in these results may be caused by inappropriate PBMT parameter settings and may not be suitable for specific species. The published dose *in vitro* or *in vivo* of other species may not be suitable for use in canine skin wound models due to the different properties of the skin and the healing process in different species [47]. It is believed that the PBMT dose recommendation for an open wound is suggested to be 2-8 J/cm^2^ [25]. However, the use of combination wavelength PBMTs has no dose setting recommendation. Therefore, the simultaneous SPMW-PBMT dosage of this study was set based on the manufacturer’s recommendations for wound healing purposes at 1-250 Hz.

Assessment of wound healing in this study was based on the macroscopic changes from a digital photograph and analyzed using image analysis software (ImageJ software^®^) as mentioned earlier, which is widely utilized in research and provides accuracy for patient monitoring in clinical practice [48]. The photographic method is accepted as an appropriate technique for measuring wound area in clinical studies without contact with the wound bed [49] and does not require histopathologic confirmation in client-owned animals [16,28,40]. The result of the current study showed a significant difference between the non-PBMT and PBMT groups from days 7 to 13 (p<0.05). At the end of the study (day 15), two cases in the L2 group had complete wound healing. This may influence the reduction rate of the wound size in the PBMT group and may result in a non-significant difference in the wound size between groups on day 15 (p=0.06).

The limitations of this study include the number of clinical cases that met the criteria and the consequent implications for the statistical power between distinct PBMT groups. This study was done on clinical cases, and the wound size at study commencement could not be controlled. Therefore, the wound size at the beginning of the study showed a statistical difference between dogs (p=0.03). The wound size needed to be adjusted because the percentage of wound area reduction inherently controls for different wound sizes at baseline before data analysis. In addition, the participants were considered healthy by general physical examination and blood profiles. Moreover, no significant differences in age (p=0.94), gender (p=0.47), and cause of wound (p=0.41) were noted. However, the variation in the individual cases may influence the wound healing process. Another limitation was the absence of histopathological results because they could not be done on the client-owned dogs. Moreover, information would be gathered if histopathological analyses were performed. Therefore, the inflammatory cell infiltration, new blood vessel formation, fibroblast formation, collagen formation, and epithelialization could not be evaluated. The possible limitation of the 830 nm PBMT is the size of the convergent probe having a small aperture of 1 cm^2^, which takes more time in treating a larger wound area. Moreover, simultaneous SPMW-PBMT is designed as a cluster probe with an aperture of 4 cm^2^, which is used for a larger treatment area, and would increase the risk of eye injuries from an incidence of reflected light. Further studies may be conducted on the potential of PBMT for wound healing in other species (e.g., cats) with different skin vascularity and healing properties. In addition, a clinical investigation into the effects of PBMT or combined blue light and PBMT on infected wounds is needed.

## Conclusion

The use of a single wavelength of 830 nm PBMT with a fluence of 4 J/cm^2^, power of 200 mW, and frequency of 50 Hz or simultaneous SPMW-PBMT (660, 875, and 905 nm) with a preset of 1-250 Hz resulted in a significant reduction in the wound area in the client-owned dogs. Therefore, PBMT could be used as an adjunctive therapy to reduce treatment duration and improve the quality of life. Further studies need to be performed to validate this result in clinical practice.

## Authors’ Contributions

NK and SJ: Designed and supervised the study. SH: Conducted the literature search and performed the experiments. SS: Performed the data analysis. NK, SJ, and SH: Wrote the manuscript. All authors read, revised, and approved the final manuscript.
